# Attractor dynamics in local neuronal networks

**DOI:** 10.3389/fncir.2014.00022

**Published:** 2014-03-20

**Authors:** Jean-Philippe Thivierge, Rosa Comas, André Longtin

**Affiliations:** ^1^School of Psychology, University of OttawaON, Canada; ^2^Department of Physics, University of OttawaON, Canada

**Keywords:** computer simulations, attractor, synchronization, oscillations, spiking neurons, mean field

## Abstract

Patterns of synaptic connectivity in various regions of the brain are characterized by the presence of synaptic motifs, defined as unidirectional and bidirectional synaptic contacts that follow a particular configuration and link together small groups of neurons. Recent computational work proposes that a relay network (two populations communicating via a third, relay population of neurons) can generate precise patterns of neural synchronization. Here, we employ two distinct models of neuronal dynamics and show that simulated neural circuits designed in this way are caught in a global attractor of activity that prevents neurons from modulating their response on the basis of incoming stimuli. To circumvent the emergence of a fixed global attractor, we propose a mechanism of selective gain inhibition that promotes flexible responses to external stimuli. We suggest that local neuronal circuits may employ this mechanism to generate precise patterns of neural synchronization whose transient nature delimits the occurrence of a brief stimulus.

## Introduction

The mammalian brain is composed of a complex network of synapses that permit the flow of electrochemical activity between populations of neurons. In the cerebral cortex, synaptic networks form a dense map whose cytoarchitecture has been studied extensively (Braitenberg and Schuz, 1998). Several factors influence the probability of local synaptic connections in cortex, including physical distance (Song et al., 2005), functional domains (sets of neurons that show similar response properties) (Mountcastle, 1997), and selective connectivity amongst similar cell types (Stepanyants et al., 2004). Another characteristic feature of cortical networks is the presence of synaptic motifs, defined as triplets (or, more generally, *n*-tuplets) of neurons whose synaptic pattern follows a particular configuration (Sporns and Kotter, 2004; Song et al., 2005; Roxin et al., 2008). These motifs provide the building blocks of connectivity at a given spatial scale, and have been explored in various contexts outside of brain connectivity, including gene regulation and other biological and artificial networks (Milo et al., 2004).

**Figure 1:**
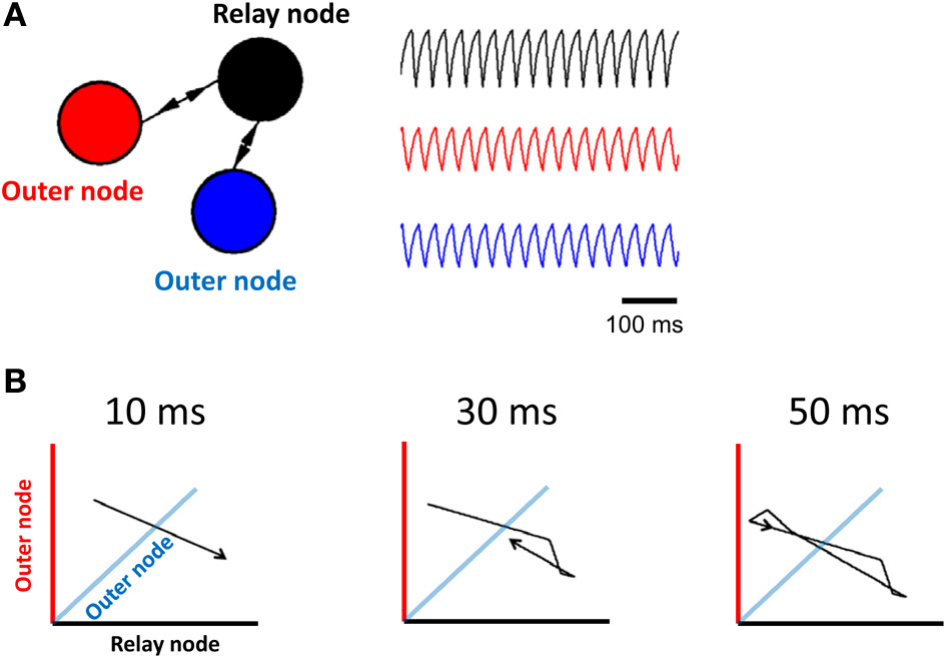
**Limit cycle activity in a simplified relay network. (A)** Illustration of a relay network, where each node is modeled by a Wilson–Cowan equation (left panel), and generates periodic activity over time (right panel). The relay node sends/receives activation to/from the two outer nodes. **(B)** 3D phase-plane plots show activity at all three nodes of the Wilson–Cowan model (axis color is associated with the corresponding population). Each plot represents activity over a short timeframe. Once the trajectory has completed a full cycle (at 50 ms), it loops back onto itself and repeats the process.

Motif configurations have been studied in the context of both local cortical networks *in vitro* (Song et al., 2005) and in the structural connectivity of macaque and cat cortex (Sporns and Kotter, 2004). In all instances, a subset of motifs reoccurs with higher-than-chance prevalence, suggesting a functional role in cortical information processing (Thivierge and Marcus, 2007). Simulated networks of neurons whose excitatory synapses follow a “relay” motif (Figure 1A)—the most frequent motif in primate visual cortex—exhibit synchronization with near-zero time lag (Traub et al., 1996; Vicente et al., 2008). This form of activity is reported in a spectrum of experiments including retinal ganglion cell recordings (Ackert et al., 2006), in cells of the lateral geniculate nucleus (Alonso et al., 1996), and in the electroreceptors of the weakly electric fish (Doiron et al., 2003). Zero-lag synchronization emerges because of the common input provided by the relay node to the two other nodes.

While computer simulations of a relay network suggest a substrate for the emergence of synchronization between neurons, these networks are limited in the scope of their behavior, and typically follow a limit cycle whose period is determined by the intrinsic properties of the model (Coombes et al., 2006; Kopelowitz et al., 2012; Viriyopase et al., 2012). This limit cycle has been shown to generalize to a large class of neuronal models that follow a relay motif (Grossberg, 1978). While cortical recordings show evidence of limit-cycle oscillations (Rodriguez et al., 1999), this behavior is typically transient in non-pathological states. Brain oscillations are usually restricted to short time periods, and remain coherent for only a limited number of cycles (Fries, 2005). Furthermore, transient neuronal responses themselves carry stimulus-relevant information in visual (Ackert et al., 2006) and olfactory (Mazor and Laurent, 2005; Geffen et al., 2009) processing. The question thus arises of how to generate transient, yet precise synchronization with connectivity that follows a relay motif, resisting the propensity of this motif to generate ongoing synchrony in a limit cycle. This question has received scant attention, despite many studies examining the impact of connectivity on simulated brain dynamics (Schuster et al., 1979; Cohen and Grossberg, 1983; Sporns and Kotter, 2004; Coombes et al., 2006; Thivierge and Marcus, 2007; Vicente et al., 2008; Goldman, 2009; Ostojic et al., 2009).

Here, we begin by examining neuronal activity in a simplified mean-field model that allows us to visualize global network activity using a phase plane plot, a graphical display of how nodes interact to produce patterns of activity. This model highlights the effect of key parameters in generating limit cycle activity, multistability, and stimulus encoding. We then turn to a second, more detailed model based on integrate-and-fire neurons, to show conditions under which a relay network leads to a strict limit cycle, thus preventing the encoding of incoming stimuli. Finally, we describe a mechanism of selective gain inhibition (Vogels and Abbott, 2009) that promotes stimuli encoding by breaking up the functional interactions in relay networks. These results carry important functional implications on how connectivity constrains patterns of neuronal activity in synaptically-coupled networks.

## Materials and methods

### Wilson–Cowan model

Our starting point is a simplified population model where the fundamental unit is a set of coupled noise-free Wilson–Cowan equations (Wilson and Cowan, 1972):

(1) $$\begin{array}{l} \varepsilon \frac{dx}{dt}=-x+\theta \text{​}\left(-wx_{\tau }+wy_{\tau }+wz_{\tau }+I\right)\\ \varepsilon \frac{dy}{dt}=-y+\theta \text{​}\left(\alpha wx_{\tau }-wy_{\tau }+I\right)\\ \varepsilon \frac{dz}{dt}=-z+\theta (\alpha wx_{\tau }-wz_{\tau }+I), \end{array}$$

where *x*, *y*, and *z* each represent the mean firing rate of a local population of neurons, *w* is a weighted connection, *I* is a constant input stimulus (set to zero by default), *α* is a free parameter (set to 1.0 by default), θ is a sigmoid function, θ(*x*) = 1/(1 + *e*^−*x*^), ε > 0 is a rate parameter that governs the speed at which the firing rate changes, and τ is a fixed synaptic transmission delay. Unless otherwise stated, connections are set to *w* = 10^3^, leading to excitatory connections between populations and inhibitory self-connections. For illustration purposes only (and bearing in mind the limited biological correspondence of this simplified account), we draw an equivalence of 1 time-step = 0.1 ms of simulated activity. Unless otherwise stated, we introduce a delay of τ = 1.5 ms in synaptic transmission from one population to another. We employ an Euler method (integration step of 0.1) for the integration of Equation 1.

### Populations of leaky integrate-and-fire neurons

In addition to the above mean-field model, we considered a network of integrate-and-fire neurons whose membrane potential is described by

(2) $$\begin{array}{l} c_{m}\frac{dV}{dt}=(V_{\text{rest}}-V)+g_{ex}(E_{ex}-V)+g_{\text{inh}}(E_{\text{inh}}-V)\\ \;+I_{\text{syn}}+R(I_{\text{ext}}+I_{\text{tonic}}), \end{array}$$

where *V*_rest_ is the resting membrane potential, *g*_*ex*_ and *g*_inh_ are synaptic conductances of excitation and inhibition, *E*_*ex*_ and *E*_inh_ are the reversal potentials of excitation and inhibition, *R* is a unit-less scalar gain, *I*_ext_ is an external current, *I*_tonic_ is a tonic current, and *c*_*m*_ is the membrane capacitance. The synaptic input *I*_syn_ for a neuron *i* is given by

(3) $$I_{\text{syn},i}=\sum_{j=1}^{N}w_{ij}K_{j},$$

where *w*_*ij*_ is a synaptic weight from neuron *j* to neuron *i*, and *K*_*j*_ is the excitatory postsynaptic membrane potential of a neuron *j*:

(4) $$K_{j}=V_{0}\sum_{s=1}^{S}\exp\text{​}\left(\frac{t_{s}-t}{\tau_{\text{fall}}}\right)-\exp\text{​}\left(\frac{t_{s}-t}{\tau_{\text{rise}}}\right),$$

where *s* = 1,..,*S* indexes spike times and *V*_0_ is a scaling factor. The rise and fall times of the postsynaptic membrane potential are given by τ_rise_ and τ_fall_, respectively. A spike is triggered when the membrane potential (Equation 2) reaches its firing threshold from below. At that point, *V* is held at 40 mV for 1 ms, then reset to −70 mV for an absolute refractory period lasting 3 ms. In all numerical simulations, we imposed a fixed time delay on synaptic transmission (see parametric values below).

Some of the above model's parameters were designed to vary across the population of simulated neurons (Thivierge and Cisek, 2008). This was achieved by randomly drawing parametric values from a Gaussian distribution with σ = 0.33 times the mean. Means for these parameters were as follows: *g*_*ex*_ (0.8 nS), *g*_inh_ (−1.5 nS), *E*_*ex*_ (0 mV), *E*_inh_ (−80 mV), τ_rise_(3 ms), τ_fall_ (5 ms), firing threshold (−55 mV), resting potential (*V*_rest_ = −60 mV) and synaptic delays (3 ms). Other parameters were constant across the entire population of neurons: *I*_tonic_ (3.5 mV), *c*_*m*_ (0.02), *R* (10), and *V*_0_ (0.09).

Synaptic connectivity (*w*_*ij*_) was configured to produce three distinct populations of neurons (with a total of 10,000 neurons per population), characterized by strong within-population interactions, and weaker between-population interactions. Both within- and between- population weights were drawn from a Gaussian distribution with mean of 100 nS (or −100 nS in the case of inhibitory neurons) and standard deviation of 0.33 times the mean. Twenty percent of connections were inhibitory. These connections were chosen randomly amongst all potential connections. Only a portion of all possible connections were present: the probability of a within-population connection between pairs of neurons was set to 0.9, while the probability of a between-population connection was set to 0.2. A cartoon illustration of three regions of neurons where between-population connections reflect a relay motif is shown in Figure 1A. Three populations of neurons are labeled by different colors, and arrows represent between-population connections.

## Results

### Network connectivity and mean-field activity

In order to investigate limit cycle activity in interconnected networks, we performed simplified simulations using a Wilson–Cowan population model (Equation 1). Activity at each node of the network was approximated by a single equation that describes mean population behavior (Figure 1A, right). We simulated a relay network for 100 s, and displayed the resulting activity on a phase plane plot (Figure 1B). This plot relates all three nodes of the network at time-step *t* vs. *t* + 1, showing a trajectory of neuronal activity. A limit cycle on a phase plane is characterized by a closed loop that repeats itself by following the same trajectory over and over again. While these simulations are highly abstracted, and represent the Wilson–Cowan equation of Equation 1 for only a specific set of parameters and initial conditions, the resulting dynamics provide a clear illustration of the influence of network connectivity on ongoing dynamics, and are in line with previous work relating relay networks with the emergence of limit cycle activity (Coombes et al., 2006; Kopelowitz et al., 2012; Viriyopase et al., 2012). Zero-lag synchronization arises here because of bidirectional connections in the relay network, allowing two nodes (in blue and red) to coordinate their activity through a third node (in black) that serves as intermediary. In this way, zero-lag synchronization arises despite the absence of direct connections between the blue and red nodes.

**Figure 2:**
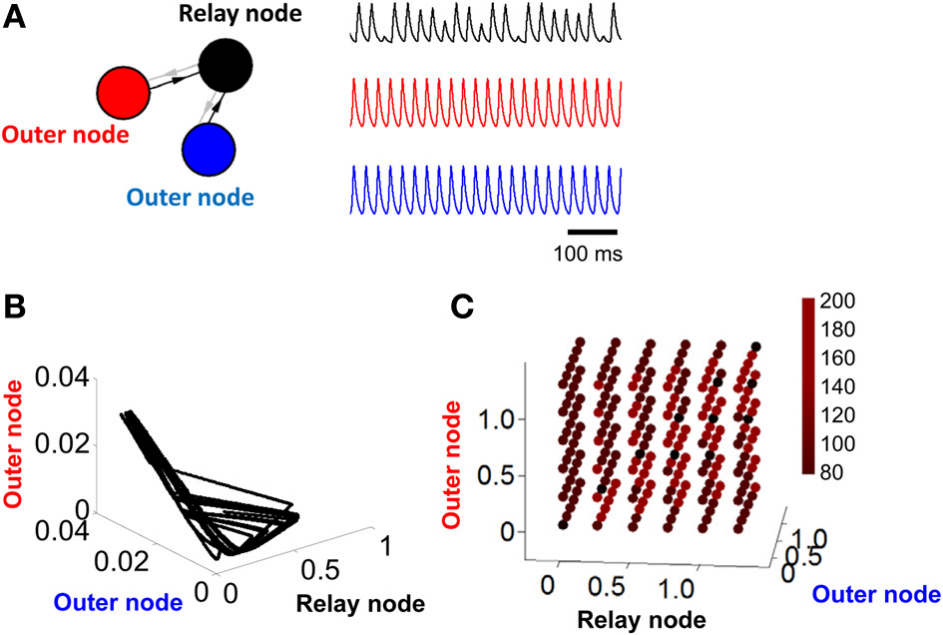
**Activity becomes unstable in a relay network with no feedback connections. (A)** Left: illustration of a three-node network where connections are strictly feedforward (connections in gray are set to zero, i.e., α = 0 in Equation 1). Right: pattern of activity obtained for each of the three nodes in **(A)** over time. **(B)** Phase-plane of activity where nodes in **(A)** are simulated with a Wilson–Cowan model. **(C)** Duration (in ms) of stable cycles in a relay network with both feedback and feedforward connections between nodes. Each dot shows initial conditions for the relay node and the two outer nodes.

In order to weaken (or remove) the limit cycle resulting from a relay network, it suffices to eliminate the influence of the relay node on the other two populations of the model (Figure 2A, left). This is done by setting α = 0 (Equation 1). In this scenario, connections are strictly feedforward, projecting onto the relay node without feedback. With this configuration, activity in two of the nodes (red and blue traces, Figure 2A, right) shows a periodic cycle; the third node (black trace, Figure 2A, right), however, shows no repeating pattern in terms of amplitude, even over extended periods of time. If we considered only the activity of the latter node, we might be led to conclude that the activity at that node is best described by random amplitude fluctuations. However, displaying the activity of the model in a phase plane reveals a hidden structure: while neuronal activity does not display a simple closed loop, it is constrained to a limited portion of the total space (Figure 2B). The activity of the model never repeats itself exactly over time, but follows an “orbit” that forms a well-defined pattern in the phase plane plot. Note that one can also change the input *I* in Equation 1 to bias the system out of a limit cycle (see Linear Stability Analysis below).

To evaluate the stability of limit cycle activity in relay networks, we let *A*_*n*_(*t*) reflect the activity of a given node *n* at time-step *t*, and sought values of *d* for which

(5) $$A_{n}(t)=A_{n}(t+kd)+\varepsilon$$

where ε was set to four orders of magnitude below the resolution of the model (ε= 10^−5^ μA) and *k* is an arbitrary constant integer. If a solution to *d* exists, the system is deemed periodic, and the value of *d* determines the duration of the period. While the exact value of this duration was dependent upon the initial conditions of the system, convergence to a limit cycle was observed across a range of starting points for *A*_1_, *A*_2_, and *A*_3_ (Figure 2C). This result shows that a relay network consistently leads to a limit cycle, with the length of the cycle dependent upon the initial conditions of the system. The finding that the length of the limit cycle depends upon initial conditions of the model is consistent with the idea of multistability in models of neuronal activity (Foss et al., 1996). Accordingly, a range of stable solutions exist, and each solution can be reached by activating the model in a particular way.

We repeated the above analysis for a network with feedforward connectivity (Figure 2A, setting α = 0 in Equation 1) and found no solution to *d* across any configuration of initial conditions. A more formal analysis of stability and of the origins of chaotic behavior in relay and feedforward networks is presented below.

**Figure 3:**
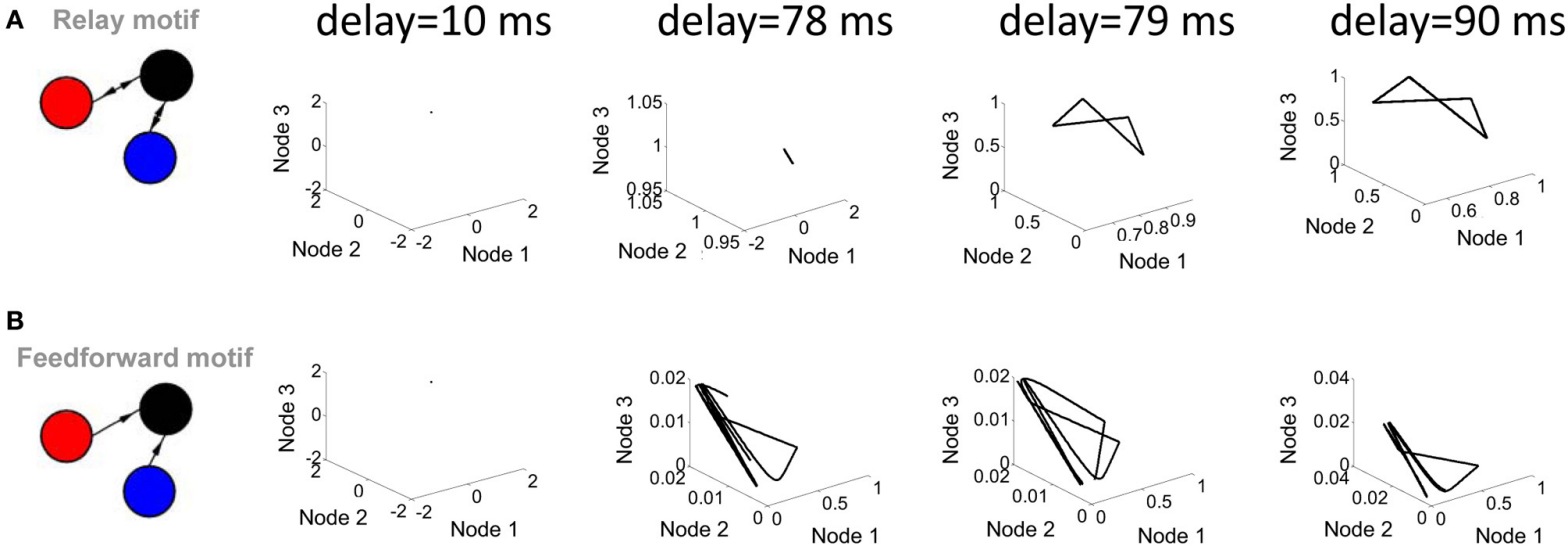
**Influence of transmission delay on network activity.** (**A**; left panel) Illustration of a relay network with both feedforward and feedback connections. (right panel) Phase-plane plots of activity simulated with a Wilson–Cowan model. Limit cycle activity emerges as a sharp transition between a delay of 78 ms and a delay of 79 ms. (**B**; left panel) Feedforward network. (right panel) Phase plane plots of activity.

Two parameters of the Wilson–Cowan model bear a strong influence on its activity. The first of these parameters is the transmission delay between nodes (the amount of time elapsed before the activity at a given node influences the activity at another node). Shorter delays (below 78 ms) did not produce limit cycle activity (i.e., no solution to Equation 5 was found); above that value, changes in the value of delays did not markedly alter the shape of the limit cycle (Figure 3A). In a strictly feedforward network, a similar result was found: short delays (e.g., 10 ms) were not sufficient to generate trajectories in the phase plane plot (Figure 3B).

**Figure 4:**
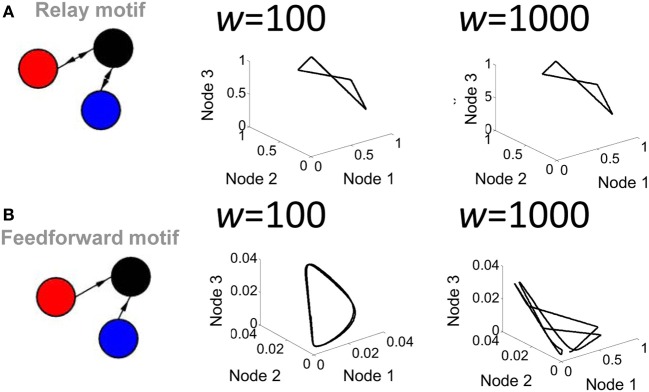
**Influence of connection strength on the activity of a relay network. (A)** Phase-plane plot of activity in a relay network where all connections had low (*w* = 100) or high (*w* = 1000) values. **(B)** Same as **(A)** but with a strictly feedforward network.

A second parameter playing a key role in network activity is the strength of the connection weights between populations. Low (*w* = 10^2^) and high (*w* = 10^3^) weights between populations yielded markedly different shapes of attractors (Figure 4). Of particular interest, a feedforward network generated a limit cycle when connection weights were low (e.g., *w* = 10^2^). In this case, the red and blue nodes oscillate and transmit that oscillation in a weak form to the black node, which then also oscillates. Together, these results show that both transmission delays and weight magnitudes influence the production of attractors in the activity of the Wilson–Cowan model.

**Figure 5:**
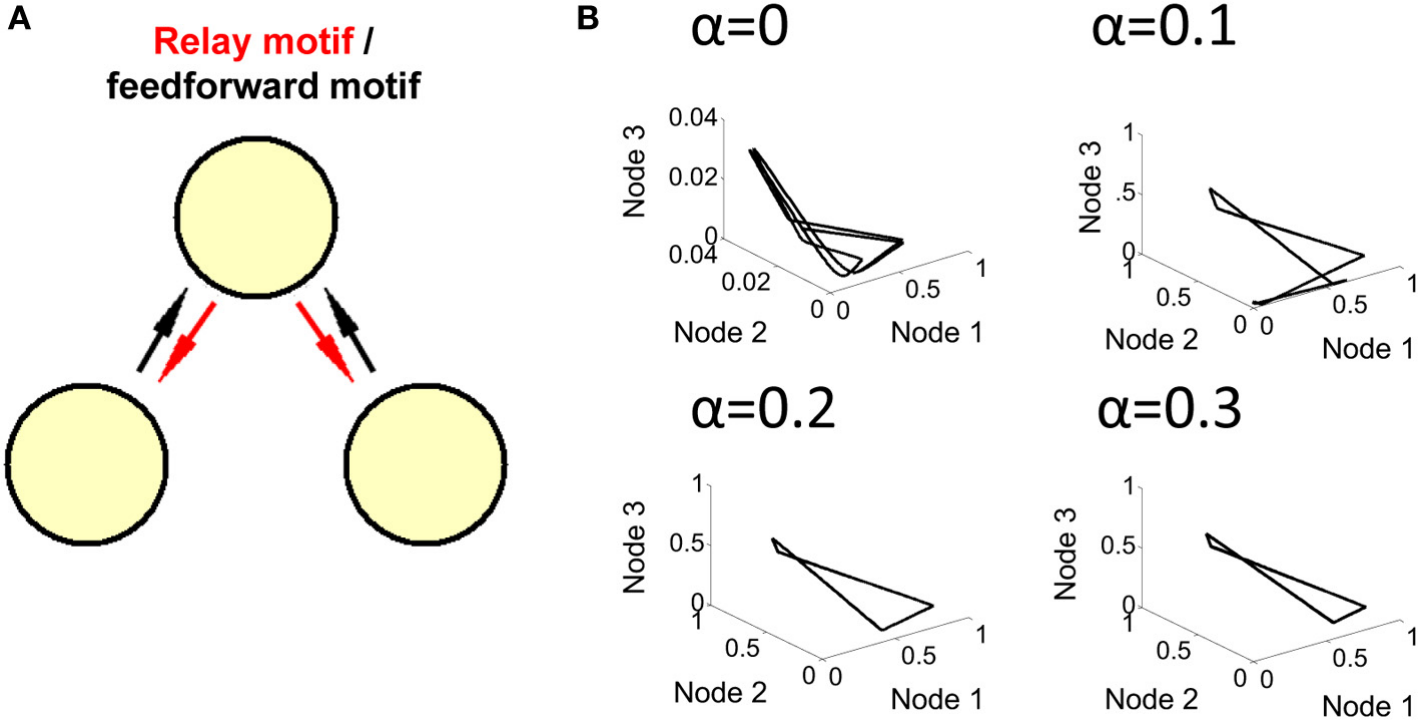
**Transition to limit cycle activity. (A)** By adjusting a single parameter α in the model (Equation 1), we can alter the strength of connections in red, generating either a feedforward network (when α = 0), or a relay network (when α > 0). **(B)** By setting the value of α to either 0, 0.1, 0.2, or 0.3 in different simulations, we found that a limit cycle emerges around a value of α = 0.2, and is maintained for higher values of this parameter. Below a value of 0.2, the network generates a more complex attractor.

To further explore the route that goes from a limit cycle to a more complex form of activity, we examined the order parameter α that modulates the influence of feedback connections from the relay node (see Equation 1). With a value of α = 0.2 and greater, feedback connections are strong enough to produce a limit cycle behavior; below that value, however, weaker feedback results in more complex forms of activity (i.e., where no solution to Equation 5 was found) (Figure 5).

**Figure 6:**
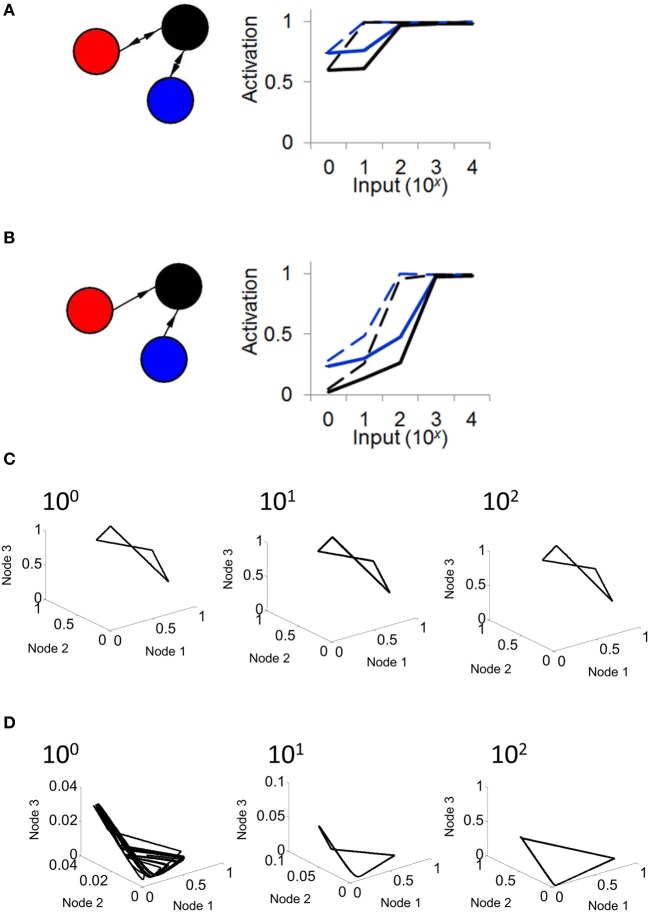
**The response of a relay network to input depends on connectivity.** (**A**; left) Illustration of a relay network with feedback connections. (right) In a relay network, mean activation increases monotonically with the strength of input, but saturates for values of input greater than 10^2^. Black and blue lines represent nodes of the network in **(A)** (activation of the red node overlaps with that of the blue node). Solid lines, weights of *w* = 1000. Dashed lines, weights of *w* = 100. (**B**; left) Network with strictly feedforward connections. (right) In a feedforward network, mean activity increases in response to input, and does not saturate until the strength of input reaches 10^3^. **(C)** In a relay network, activity follows a limit cycle regardless of the strength of input. **(D)** In a feedforward network, activity follows a limit cycle for stronger input (10^2^) but not for weaker input (10^0^).

Next, we examined the response of a Wilson–Cowan model to a constant input injected into all three nodes. In different simulations, each lasting 100 s, we varied the intensity of input (from *I* = 0,…,10^4^). When connectivity followed a relay network, activity in the network increased in response to inputs ranging from *I* = 0 to *I* = 10^2^, then saturated from *I* = 10^2^ to *I* = 10^4^ (Figure 6A). This result was found with both high (*w* = 10^3^) and low (*w* = 10^2^) connection strength. We compared these results with those obtained when injecting input into a network with feedforward connectivity. In this case, activity monotonically increased in response to inputs from *I* = 0 to *I* = 10^3^, a broader range than that obtained with a relay network (Figure 6B). Upon close inspection, the difference in responses between the relay and feedforward networks is largely explained by the fact that the feedforward network exhibits lower activation under weak input (i.e., mean activation is low when *I* is small). To further probe the effect of input on network dynamics, we examined phase plane plots of activity, as described above. In relay networks, for all values of input tested, activity consistently yielded a limit cycle (i.e., where a solution to Equation 5 could be found) (Figure 6C). By contrast, in feedforward networks, activity yielded different patterns depending on the intensity of input. With weak input (*I* < 10), activity followed no repeating trajectory (Figure 6D); however, as the intensity of input increased, network activity settled into a limit cycle attractor. In sum, a strictly feedforward network led to a greater dynamical range of responses than a relay network; in addition, a feedforward network resulted in a different attractor depending on the strength of input, whereas a relay network always resulted in a limit cycle attractor.

**Figure 7:**
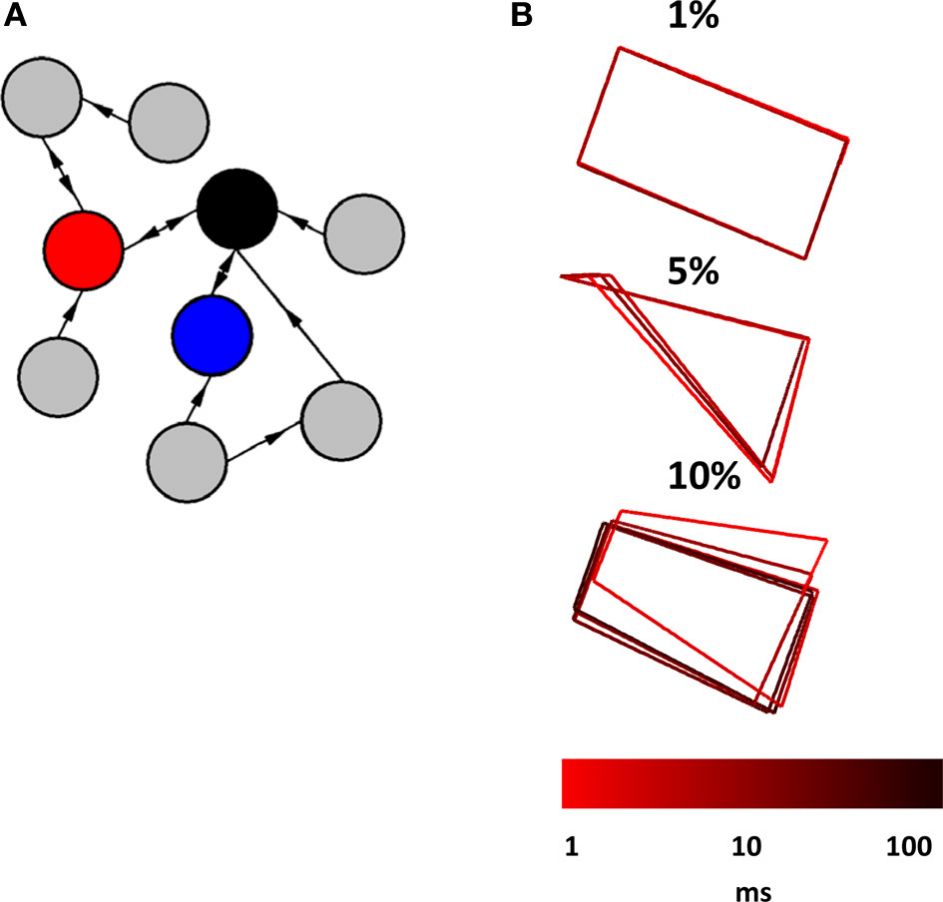
**Relay network embedded in a broader network of randomly interconnected nodes. (A)** Cartoon illustration of a relay network (red, blue, and black nodes) embedded in a larger network that has random connectivity (gray nodes). The actual network that we simulated had a total of 1000 nodes (not counting those of the relay network itself). **(B)** Phase-plane plots showing the activity of a relay network. This activity is characteristic of a limit cycle where perturbations from ongoing activity in the surrounding network provide slight alterations to the trajectory. Three plots show patterns of activity obtained from different probabilities of connection between nodes in the surrounding network (1, 5, and 10%). Each run of the model lasted 100 s at a resolution of 0.1 ms.

In a final series of simulations, we considered a scenario where a relay network is embedded in a larger network of Wilson–Cowan nodes. We began by generating a sparse randomly connected network of 1000 nodes, where one node had a 1% probability of being connected to any given node in the network. Then, we selected three nodes at random and forced their connectivity to follow a relay network (Figure 7A). All connection weights, both within the relay network and outside of it, were set to *w* = 10^3^ if a connection was present, and *w* = 0 otherwise (self-connections were set to *w* = −10^3^). Examples of activity generated when a relay network was embedded in a larger network are shown in Figure 7B. The resulting pattern of activity can be described as a “noisy” limit cycle, where perturbations coming from activity in the surrounding network made the trajectory of the limit cycle deviate from its path. Here, embedding a relay network in a larger network did not result in a fundamentally different pattern of activity, but rather a perturbed version of the original pattern obtained when the relay network was simulated as a stand-alone network. Of course, increasing the density of connections within the larger network would lead to more pronounced perturbations, yet would result in a less plausible scenario from the point of view of cortical connectivity. Excitatory cortical cells receive only sparse afferents from other excitatory cells. The probability of contact between two neocortical excitatory cells that are 0.2–0.3 mm apart is estimated to be *p* < 0.1, and between two such cells that are more than 1 mm from each other, *p* < 0.01 (Braitenberg and Schuz, 1998; Song et al., 2005). Because nodes in the Wilson–Cowan are aimed at simulating populations of neurons rather than individual synaptic contacts, we rely on the latter probability as a point of comparison. Our simulations of three-node relay networks embedded in larger random networks show that patterns of activity are robust to the influence of ongoing activity generated from the surrounding network under reasonable conditions of connectivity.

### Linear stability analysis

The above simulations show that relay networks are prone to oscillations that are caught in a limit cycle, while feedforward networks generate more complex forms of activity that do not oscillate in a strict manner. Here, we derive a linear stability analysis that yields insight into the propensity of a relay network to oscillate compared to a feedforward network. We consider the three node (*x,y,z*) model of Equation 1, where outer nodes *y* and *z* (red and blue nodes, Figure 1A) project to a relay node *x* (black node in Figure 1A) with connection weight *w* > 0. Likewise, node *x* projects back to nodes *y* and *z*, but with a connection strength αw. Here α is an adjustable parameter between 0 and 1; when α = 1, the three-node system embodies a relay network, while for α = 0 it represents a feedforward network. This formulation enables us to smoothly move between a relay and feedforward network. It also enables us to investigate all system parameters, combinations of parameters, and initial conditions.

The fixed points of the system in Equation 1, that is, the values of (*x,y,z*) for which the derivatives are zero, are given by solutions of the following non-linear equations:

(6a) $$x^{*}=\theta \text{​}\left(-\omega x^{*}+\omega y^{*}+\omega z^{*}+I\right)$$

(6b) $$y^{*}=\theta \text{​}\left(\alpha \omega x^{*}-\omega y^{*}+I\right)$$

(6c) $$z^{*}=\theta \text{​}\left(\alpha \omega x^{*}-\omega z^{*}+I\right).$$

To solve the above system, we first note that the solutions remain invariant upon interchanging *y* and *z*. The same can be said of the original system (Equation 1). This means that a solution (*x*(*t*),*y*(*t*),*z*(*t*)) will be the same as a solution (*x*(*t*),*z*(*t*),*y*(*t*)), provided that the variables *y* and *z* have the same initial values; in other words, *y*(*t*) tends to *z*(*t*) when time is large enough provided that *y*(0) = *z*(0). As for the fixed point, it will be such that y^*^ = z^*^, that is, it will lie on the plane (x^*^,y^*^,y^*^). Numerical simulations indeed reveal that this is the case, and also that solutions evolve to ones where *y* approaches *z* for a large range of differences between *y*(0) and *z*(0). However, solutions where *y*(0) differs significantly from *z*(0) can evolve such that both solutions are the same, but maintain a fixed time lag between them. One should note that the value of the input *I* determines the precise value of the fixed point. In other words, this system admits a simple rate coding where the system settles onto a fixed point “rate” that varies smoothly with the strength of the input. As we will see below, as certain parameters change, this system undergoes a “Hopf bifurcation,” or in other words a transition between a stable fixed point—corresponding to a stable constant firing rate in the model—to stable limit cycle oscillations of the firing rate. Our paper mainly concerns the robustness of these oscillations.

In order to investigate the dynamical properties of the system, in particular what combinations of parameters lead to an equilibrium (i.e., a fixed point) or an oscillation, an important starting point is to non-dimensionalize the equations. This will be done here by scaling the time variable by the delay, leading to a new continuous dimensionless time *T* = *t*/τ that is counted in the number of delays (e.g., *T* = 5.677 means *t* = 5.677τ). Further defining *k* = ε/τ, and new variables *X*(*T*) = *x*(*t*), *Y*(*T*) = *y*(*t*), and *Z*(*T*) = *z*(*t*), the model evolves according to:

(7a) $$\begin{array}{l} k\frac{dX}{dT}=-X(T)+\theta [-wX(T-1)+wY(T-1)\\ \;+wZ(T-1)+I] \end{array}$$

(7b) $$k\frac{dY}{dT}=-Y(T)+\theta [\alpha wX(T-1)-wY(T-1)+I]$$

(7c) $$k\frac{dZ}{dT}=-Z(T)+\theta [\alpha wX(T-1)-wZ(T-1)+I].$$

The fixed point (X^*^,Y^*^,Z^*^) for this system is identical to that of Equations 6a–c above, with the substitution of *X,Y,Z* for *x,y,z*. While it is not possible to explicitly solve this transcendental system, our numerical simulations reveal that there is only one relevant fixed point (X^*^,Y^*^,Z^*^). Investigating the linear stability of this fixed point will reveal how solutions behave near this point, and in particular, if bifurcations can occur between a stable equilibrium and a stable oscillation. This linearization is done using a multivariate Taylor expansion, keeping only the first order terms. We first move the origin (0,0,0) onto the fixed point (X^*^,Y^*^,Z^*^) by a change of coordinates: *X*′ = *X* − X^*^, *Y*′ = *Y* − Y^*^, *Z*′ = *Z* − Z^*^. The resulting linearized system is given by:

(8a) $$\begin{array}{l} k\frac{dX^{\prime }}{dT}=-X^{\prime }(T)-wAX^{\prime }(T-1)+wAY^{\prime }(T-1)\\ \;+wAZ^{\prime }(T-1) \end{array}$$

(8b) $$k\frac{dY^{\prime }}{dT}=-Y^{\prime }(T)+\alpha wA^{\prime }X^{\prime }(T-1)-wA^{\prime }Y^{\prime }(T-1)$$

(8c) $$k\frac{dZ^{\prime }}{dT}=-Z^{\prime }(T)+\alpha wA^{\prime }X^{\prime }(T-1)-wA^{\prime }Z^{\prime }(T-1),$$

where $A={\frac{d\theta (g)}{dg}|}_{g^{*}}$ with g^*^ = −wX^*^ + wY^*^ + wZ^*^ + *I*, and $A^{\prime }={\frac{d\theta \left(h\right)}{dh}|}_{h^{*}}$ with h^*^ = α wX^*^ − wY^*^ + *I*. Both *A* and *A*′ are slopes of the firing function, and act as a feedback gain. One observation that can be made from the analysis thus far is that, in order to examine the linear properties of either the relay or the feedforward networks, the only important parameters are the ratio *k* and the products *wA* and *wA*′.

A full analysis of this system is beyond the scope of our needs here, but we will make a few observations. First, this system can be simplified further by defining two new variables as the sum *S* and difference *D* of *Y*′ and *Z*′, *S* = *Y*′ + *Z*′ and *D* = *Y*′ − *Z*′. This yields the system

(9a) $$k\frac{dX^{\prime }}{dT}=-X^{\prime }(T)-wAX^{\prime }(T-1)+wAS^{\prime }(T-1)$$

(9b) $$k\frac{dS}{dT}=-S(T)-wA^{\prime }S(T-1)+2\alpha wA^{\prime }X^{\prime }(T-1)$$

(9c) $$k\frac{dD}{dT}=-D(T)-wA^{\prime }D(T-1).$$

In the (*X*′,*S*,*D*) coordinates, it becomes apparent from Equation 9c that the difference between the activities of the two nodes *y* and *z* behaves independently of the variables *X*′ and *S*; these latter two variables, however, evolve in a coupled manner. It is known that, since *w* > 0, the difference *D* obeys linear delayed negative feedback dynamics (Erneux, 2009); consequently, if either (or both) the delay or the linear connection weight *wA*′ are sufficiently large, then the stable fixed point will continuously transition into a stable oscillation (also known as a stable limit cycle). In technical terms, this process is termed a supercritical Hopf bifurcation.

Assuming a relay network (α = 1) and the stable fixed point case, the activities at nodes *y* and *z* will be constant and equal after a transient period (leading to a trivial form of zero-lag synchrony). This is because D^*^ = 0 implies that *Y*′ and *Z*′ are at their equilibrium values of 0, that is, *Y* = Y^*^ and *Z* = Z^*^ (recall that Y^*^ = Z^*^). This implies that one can effectively study the dynamics of the whole model by focusing on Equations 8a,b alone.

For the feedforward network (α = 0) and the stable fixed point case, it is clear already from Equations 8b to 8c that the behavior of *Y*′ and *Z*′ will be the same, up to a time shift that depends on their initial conditions. In fact, even considering the full dynamics in Equations 1a–c, it is seen that, for the feedforward network, both variables *y* and *z* have the same rule governing their evolution, but behave independently of each other. Further, *y* and *z* are merely a source of external forcing on node *x*. If the parameters are such that *y* and *z* tend to a fixed point y^*^ = z^*^, then over long periods of time node *x* will receive an identical constant forcing from each of these nodes. Node *z* could be eliminated, and the weight of the connection from node *y* doubled—node *x* would not see the difference (the same holds true if replacing *y* by *z*).

Alternately, in a feedforward network with α = 0, the parameters can be such that *y* and *z* oscillate autonomously. Their sum in Equation 1a also oscillates at the same period, and qualitatively, the dynamics of node *x* amounts to a periodically-driven delay-differential equation. The dynamics can be very rich in this case, with chaotic solutions and/or long transients, since the unforced system *x* can oscillate on its own, and this oscillation competes with the one imposed by the sum of *y* and *z*. This is the kind of solution we find in the feedforward network (see Figure 2A).

Coming back now to a relay network (α = 1), but this time with an oscillation for the difference variable *D* in Equation 9c, variables *Y* and *Z* will move close and away from each other periodically. This case also potentially leads to a complex solution. But for the parameters of interest in our study, the feedback from *x* to nodes *y* and *z* has a stabilizing effect, in the sense that the whole three-dimensional system usually settles on a limit cycle where all nodes oscillate at the same frequency.

One can carry out a linear stability analysis to find the regions of parameter space where a Hopf bifurcation occurs, using the reduced *X-S* system of Equations 9a,b. One first substitutes trial solutions *x*(*t*) = *x*_*o*_exp(λ*t*) and *y*(*t*) = *y*_*o*_exp(λ*t*) into Equations 9a,b, where λ = μ + *i*ω is a complex eigenvalue (note that we denote the angular frequency by ω, distinct from the coupling weight *w*). Assuming this solution is valid for arbitrary non-zero constant amplitudes *x*_*o*_ and *y*_*o*_, and defining the effective feedback gains β = *wA* and β ' = *wA*′, this yields the characteristic equation

(10) $$(k\lambda +1+\beta e^{-\lambda })(k\lambda +1+\beta^{\prime }e^{-\lambda })=2\alpha \beta \beta^{\prime }e^{-2\lambda }.$$

This equation admits an infinite number of complex conjugate roots (i.e., values of *λ*) corresponding to eigenvalues for the system of Equations 9a,b linearized around the fixed point. In order to find the conditions where the roots migrate from the left hand side to the right hand side of the complex plane (a characteristic of a Hopf bifurcation) we set the real part of the eigenvalue to zero: μ = 0, i.e., λ = *i*ω in Equation 10**. The resulting two equations obtained by setting both the real and imaginary parts of this special form of Equation 10 equal to zero define a relationship between all the parameters and the frequency ω at the onset of oscillation.

With respect to the Hopf bifurcation, the feedforward case (α = 0) is well-documented (Erneux, 2009). In particular, a bifurcation occurs when increasing either the delay or β; the higher the one is, the smaller the required value of the other in order for the Hopf bifurcation to occur (if both parameters are high, the system is clearly in the oscillation regime). From the first factor on the left hand side of Equation 10, the frequency of the zero-amplitude solution born at the bifurcation is given by $\omega =\sqrt{\beta^{2}-1}/k$, with β being the first root of $\tan[\sqrt{\beta^{2}-1}/k]=-\sqrt{\beta^{2}-1}$. The same expressions but with β' substituted for β apply to the second factor on the left hand side of Equation 10. Numerically, we find that the *X* system starts oscillating when the coupling strength is *w* ≈ 9.15 with the delay fixed at 1. At this onset, the *S* system still goes to a fixed point. This is so because, as the coupling strength *w* increases, the first factor acquires a purely imaginary root before the second factor does. This situation where *X* oscillates but *S* does not is possible because of the unidirectional coupling from *S* (i.e., *Y* and *Z*) to *X*.

In the relay case (α > 0), the analysis of the roots of Equation 10 is much more involved and beyond the scope of this paper. Numerically, we find that even for very small values of α, choosing *w* ≈ 9.15 as in the previous paragraph now yields an oscillation in *S*, and a larger amplitude oscillation in *X*. Both the *X* and *S* oscillations are at the same frequency, i.e., it is a global oscillation of the whole bi-directionally coupled system. In other words, a smaller delay or effective feedback gain *A* or *A*′ can then generate oscillatory activity. Thus, based on the transition between a fixed point and a limit cycle, the relay network is more prone to oscillate when compared to the feedforward network.

In summary, our stability analysis reveals that both the relay and feedforward networks can exhibit a Hopf bifurcation. Transitions to the limit cycle are favored by an increase in two parameters: the delay, or the product of the connection weight and the slope of the firing function evaluated at the fixed point (the parameters *k* and *I* also have an effect, but this was not explored here). Upon increasing α we find that the network is more prone to oscillate (c.f., Figures 1A, 2A). In the feedforward case, when the outer nodes (red and blue nodes in Figure 2A) are in a limit cycle regime, the third node (black node in Figure 2A) produces complex dynamics via the interaction of its intrinsic oscillation and the unidirectional periodic forcing from the two outer nodes. This system can be largely understood with only one variable instead of three. By increasing the value of α, we transition from a feedforward to a relay network where the dynamics of the whole system settle onto a limit cycle.

Taken together, simulations and analysis of the Wilson–Cowan model show a key role of delays, connection strength, and relay connectivity on the ability of the model to generate limit cycle activity. Further, results show that a feedforward network yields a broader range of responses to stimuli than a relay network. While these results reveal that the elimination of feedback projections from the relay node to the outer nodes can break the network out of a limit cycle, it is unclear how this could be achieved in living synaptic networks. In the next section, we explore a mechanism by which selective inhibition of relay neurons provides a network with the ability to escape its limit cycle and modulate its activity in response to incoming stimuli.

### A relay network with spiking neurons

**Figure 8:**
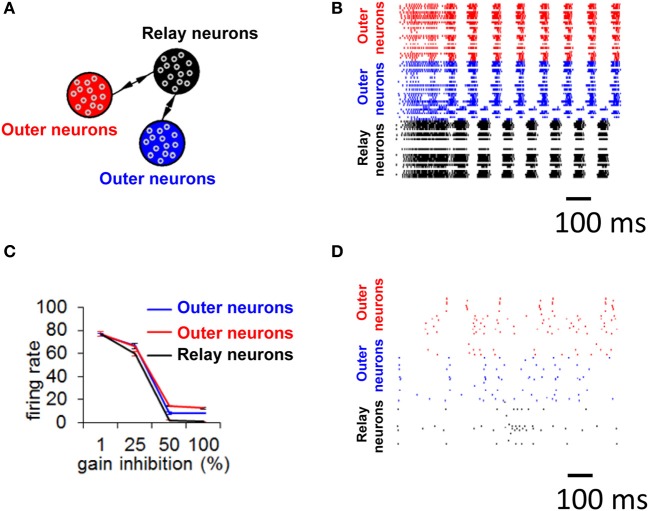
**Selective gain inhibition prevents limit cycle activity. (A)** Relay network, with neurons divided into three subpopulations (shown in red, black, and blue, see Materials and Methods). Arrows indicate the presence of between-population connections. **(B)** Spike raster of spontaneous activity for 100 neurons from the relay network in **(A)**, with gain inhibition set to its default value (*g*_inh_ = 1.5 nS). **(C)** Influence of gain inhibition on mean firing rates across a whole network. Each value of the graph is obtained from a simulation where we increased gain inhibition above its default value (1.5 nS) by a given percentage for neurons of the population in black **(A)** while gain inhibition for the other two populations was held at its default value. This increased gain inhibition reduces the excitatory coupling from the relay neurons (black) to other populations. **(D)** Spike raster for 100 neurons of relay network from **(A)**, with gain inhibition set to *g*_*inh*_ = 2.25 nS (corresponding to a 50% increase from baseline).

We now turn to a more detailed model of neuronal activity based on 30,000 integrate-and-fire neurons divided into three distinct populations, resulting in a global connectivity that followed a relay network (Figure 8A, see Materials and Methods) (Thivierge and Cisek, 2008, 2011; Rubinov et al., 2011). Simulated activity in this network (Figure 8B) shows the appearance of a global limit cycle, with two of the populations (in red and blue) exhibiting synchronization with near-zero time-lag (Vicente et al., 2008). This pattern of activity never faded away for as long as the simulation was carried out (in this case, 5 min). Extensive simulations revealed that the emergence of a limit cycle was not sensitive to initial conditions of the network. This result mirrors those obtained with the mean field model described above. Notice, however, that while the overall pattern of activity in the network follows a limit cycle, the precise spike times of individual neurons do not, because of intrinsic fluctuations in the model (no external noise was added, see Equation 2). In addition, notice that not all neurons are synchronized, and some of the neurons remain quiescent throughout.

**Figure 9:**
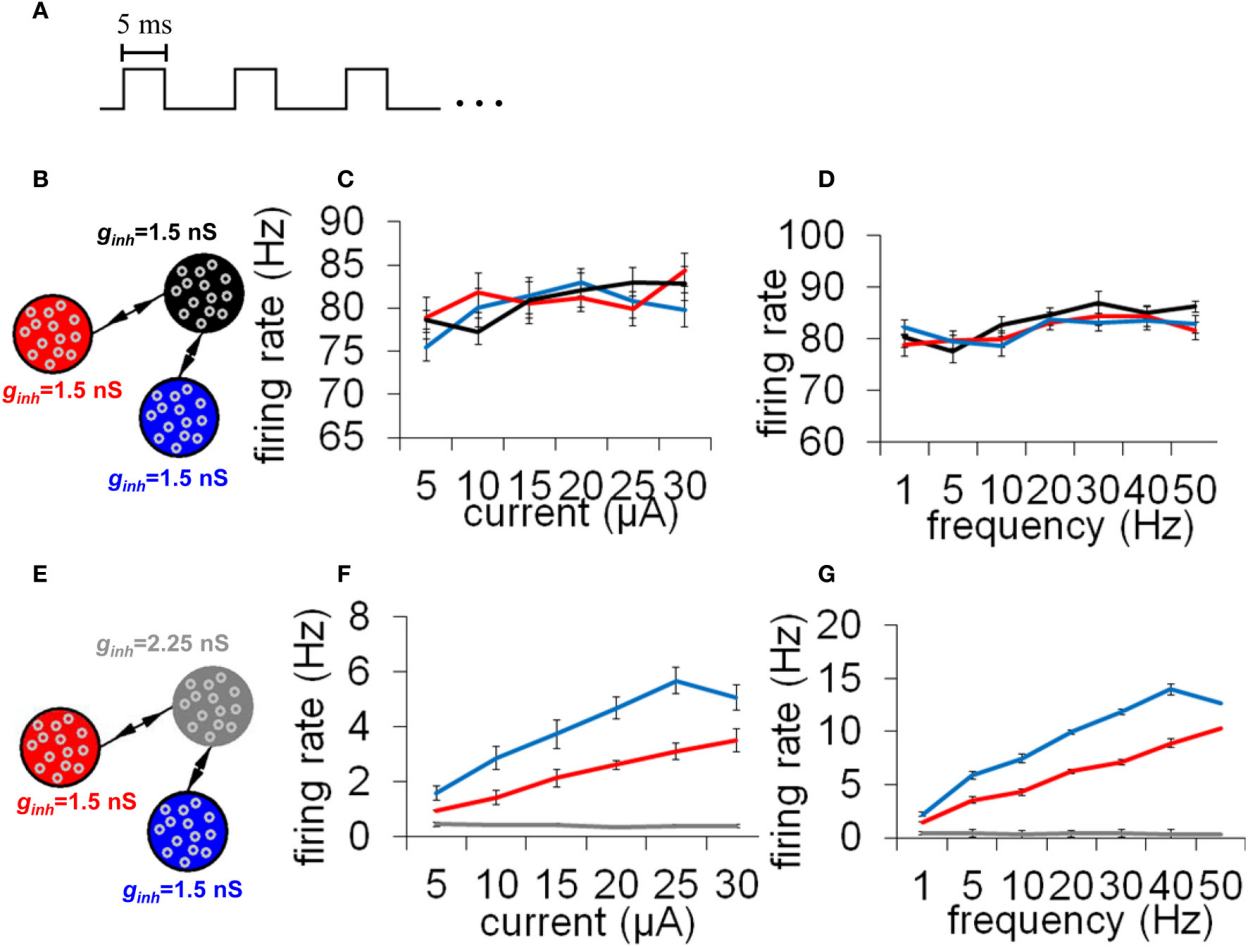
**Responses to a periodic stimulus delivered to all neurons of an integrate-and-fire network. (A)** Temporal evolution of the stimulus. All stimuli had pulses lasting 5 ms each; different stimuli varied according to the frequency (Hz) and amplitude (μA) of these pulses. **(B)** Network with a relay configuration. The network was composed of three subpopulations of neurons, each having gain inhibition set to *g*_inh_ = 1.5 nS. **(C,D)** Response to external stimuli in a relay network. We monitored mean firing rates within each population of neurons in response to different current intensities **(C)** and stimulus frequencies **(D)**. **(E)** Network where one subpopulation of neurons (the “relay neurons”) had gain inhibition set to *g*_inh_ = 2.25 nS (a 50% increase from the default value). **(F,G)** Responses to external stimuli in a relay network with increased gain inhibition as shown in **(E)**. All simulations were run for a total of 30 s of activity. Vertical bars = SEM.

While precise synchronization may convey information about the input to a neuronal circuit (Thivierge and Cisek, 2008), we argue that a strict limit cycle imposes severe constraints on the behavior of circuits in response to an incoming stimulus. Consider a periodic stimulus that is delivered at a fixed square-pulse voltage (width of 5 ms) across all neurons (Figure 9A). By varying the inter-stimulus interval and voltage intensity, we can examine conditions under which network activity is modulated by the incoming stimulus. Simulated network activity was generated for 30 s and a periodic stimulus was delivered during that entire time to all neurons. A network configured according to a relay network (Figure 9B) exhibited only marginal modulations in mean firing rate in relation to either the intensity (Figure 9C) or the frequency (Figure 9D) of stimulation. This rigid behavior is explained by the fact that a relay network is highly entrenched in limit cycle activity (Figure 8B); this activity cannot easily be dislodged from this attractor by incoming stimuli. Put differently, a system that has reached a state of global oscillation cannot easily be affected by external perturbations (Golubitsky et al., 2006).

### Selective gain inhibition

A synaptic configuration based on a relay network is highly prevalent in mammalian cortex (Sporns and Kotter, 2004; Song et al., 2005), yet the above simulations show that such a network promotes the emergence of a limit cycle where activity is largely unaffected by an incoming stimulus. To reconcile these observations, one possibility is that cortical neural circuits are capable of dynamically reconfiguring their pattern of functional interactions such that an architectural substrate based on a relay network could disengage from its strict limit cycle behavior and generate more flexible responses to incoming stimuli.

It is unclear, however, how biological circuits may be able to disengage from strict limit cycle activity. Under the reasoning that relay neurons (in black, Figure 8A) are responsible for driving zero-lag synchrony, we suggest that if we tune down the influence of that subpopulation, we may prevent the emergence of a global attractor. There are several ways in which this could be achieved; here, we describe one candidate mechanism based on selective gain inhibition (Vogels and Abbott, 2009). By tuning up the inhibitory gain (*g*_inh_, Equation 2) of relay neurons, we can selectively reduce activity in these neurons. In turn, less activity would flow from the relay neurons to other neurons in the model, thus altering the global patterns of neuronal activity.

To test the idea of selective gain inhibition, we simulated spontaneous activity in a network with a global connectivity based on a relay network, and, in different simulations, applied gradually increasing values of gain inhibition to relay neurons (Figure 8C). When gain inhibition was increased by 50% from its baseline value (from 1.5 to 2.25 nS), network activity was no longer characterized by synchronized activity (Figure 8D compared to 8B). Spontaneous activity in this regime yielded an overall low firing rate (mean rate of 1.01 Hz, s.d. 0.44) and followed no strict repeating pattern over time. Importantly, a balance of gain inhibition was necessary: if inhibition was too low (<50% increase from baseline), network activity remained comparable to baseline (Figure 8C). Conversely, if gain inhibition was too high (100% increase from baseline), activity in the relay population vanished completely.

To examine the effect of selective gain inhibition on a network's response to an incoming stimulus, we began with a network whose global connectivity follows a relay network, as described earlier. Then, we increased gain inhibition by 50% in all of the relay neurons (Figure 9E). In response to increasing stimulus intensities, the two neuronal populations sending input to the relay neurons modulated their mean firing rate in a near-monotonic fashion (Figure 9F). The same two populations also increased their firing rate in proportion to increased stimulus frequency (Figure 9G). A network with increased gain inhibition was thus able to modulate its firing rate based on an incoming stimulus, and did not remain stuck in a persistent state of activity. Put differently, increased inhibition in this circuit resulted in increased responsiveness to stimuli.

**Figure 10:**
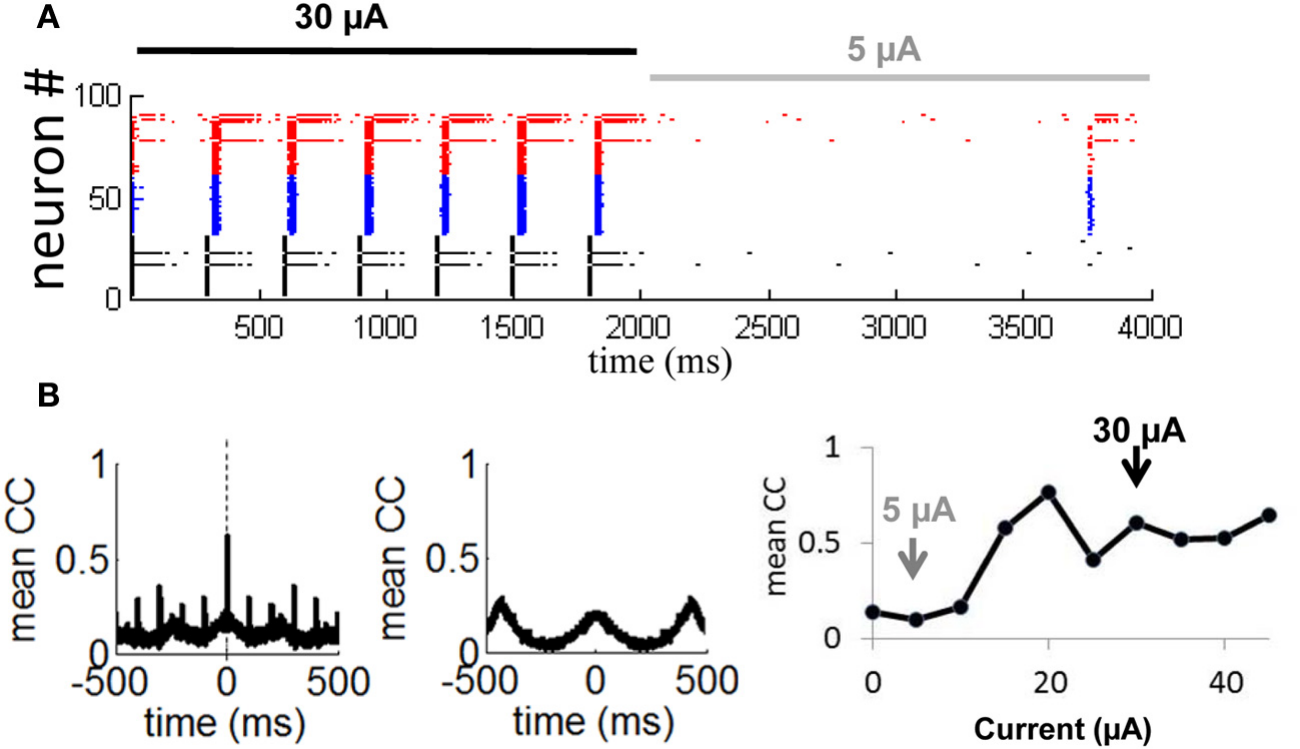
**Transient synchronization in response to stimulation. (A)** Spike raster showing responses of 100 neurons from a relay network to a strong external current (*I*_ext_ = 30 μA, solid black line) followed by a weak current (*I*_ext_ = 5 μA, solid gray line). Gain inhibition of the relay neurons was set to *g*_inh_ = 2.25 nS (50% higher than baseline) throughout the simulation. **(B)** Mean cross-correlation of the network during presentation of a strong current (left panel) and a weak current (middle panel). Right panel: Mean cross-correlation as a function of external current. Black and gray arrows show a weak (*I*_ext_ = 5 μA) and strong (*I*_ext_ = 30 μA) current as simulated in **(A)**. Vertical dashed line: zero time-lag.

In follow-up simulations, we injected a network having selective gain inhibition (*g*_inh_ = 2.25 nS) with a stimulus consisting of a constant current (30 μA) lasting 2000 ms. After that time, the stimulus was reduced to 5 μA and held constant for 2000 ms (Figure 10A). During presentation of the first stimulus (30 μA), activity was highly synchronized and strongly periodic. As soon as the first stimulus ended and the second stimulus (5 μA) began, the network became quiescent. Neurons thus produced a highly synchronized and periodic response to a stronger stimulation, and relatively little response to a weaker stimulation. This simulation shows the capacity of a network with selective gain inhibition to generate synchrony based on stimulus amplitude. Such transient responses would not be possible without selective gain inhibition, given that a network configured with a relay network follows a persistent limit cycle attractor (Figure 8B) and does not modulate its response to incoming stimuli (Figures 9C,D).

To further examine the transient synchronization of a network in response to a stimulus, we designed, as above, a relay network where we increased the gain inhibition of the relay population of neurons (Figure 8A, in black) by 50% from its baseline value (from 1.5 to 2.25 nS). We then injected a constant stimulus of 30 μA into all neurons for a 10 s period. We computed the cross-correlation between each pair of neurons during the stimulus presentation:

(11) $$C_{ij}=\frac{E\text{​}\left\{[x_{i}\text{​}\left(t\right)-E_{i}]\left[x_{j}\text{​}\left(t\right)-E_{j}\right]\right\}}{\sqrt{E\text{​}\left\{{\left[x_{i}\text{​}\left(t\right)-E_{i}\right]}^{2}\right\}E\text{​}\left\{{\left[x_{j}\text{​}\left(t\right)-E_{j}\right]}^{2}\right\}}},$$

where *x*_*i*_(*t*) and *x*_*j*_(*t*) are the time-series of two given neurons having means *E*_*i*_ and *E*_*j*_, respectively. Next, we obtained a cross-correlogram of activity by taking the mean cross-correlation across all pairs of neurons. We found prominent zero-lag synchronization (Figure 10B, leftmost panel, vertical dashed line), as typical of activity for relay networks (Vicente et al., 2008). Hence, selective gain inhibition did not disrupt the capacity of a relay network to generate zero-lag synchronization. When we repeated the above simulation with a weaker input current (5 μA), cross-correlations no longer displayed a prominent peak at zero time-lag as obtained with a stronger stimulation of 30 μA (Figure 10B, middle panel). Selective gain inhibition thus prevented a relay network from spontaneously generating zero-lag synchronization.

In a final series of simulations, we injected a 10 s input of various intensities (from 0 to 45 μA) into a relay network with selective gain inhibition as described above. For each input intensity, we computed the mean zero-lag cross-correlation across all pairs of neurons. This value increased as the input intensity was gradually amplified from 0 to 45 μA (Figure 10B, rightmost panel, gray and black arrows), then remained stable from 30 to 45 μA. The network thus modulated its degree of zero-lag synchronization in response to inputs of various current intensities, within a given range.

Taken together, our results show that selective gain inhibition can modulate the behavior of a relay network, such that the network can generate zero-lag synchronization in response to an incoming stimulus, yet does not remain stuck in a global attractor dominated by a fixed limit cycle.

## Discussion

While there is a growing consensus that patterns of structural connections in the brain provide the backbone for a rich repertoire of activity (Bullmore and Sporns, 2009), here we argue using both simulations and mean-field analysis that a relay network imposes strict constraints on the types of dynamics produced by a network. Going further, simulation results using spiking neurons suggest that a mechanism of selective gain inhibition allows a network to modulate its patterns of activity and escape the rigid constraints imposed by synaptic connectivity, providing flexible and transient responses to an incoming stimulation.

While there are several examples of transient zero-lag synchronization in the central nervous system, a prominent one is found in the response of direction-sensitive (DS)—ON ganglion cells in the visual system (Ackert et al., 2006). In these cells, GABAergic inhibition forces activity to desynchronize following a transient phase of stimulus-induced zero-lag synchronization initiated by gap junction couplings between DS-ON and wide-field amacrine cells. While GABAergic inhibition suppresses zero-lag synchronization, it leaves intact the broad synchronization profile of cross-correlations at non-zero time lags. An analogous behavior was observed in our simulated spiking neurons, where selective gain inhibition suppresses stimulus-induced zero-lag synchronization (Figure 10B, leftmost panel) but leaves intact the broad profile of cross-correlations (Figure 10B, middle panel). Zero-lag synchronization amongst neighboring DS-ON cells is the product of shared excitation passing exclusively through an indirect gap junction coupling that operates through polyaxonal amacrine cells. Similarly, in simulations of spiking neurons, zero-lag synchronization emerges between two populations of neurons that are coupled exclusively through an indirect excitatory pathway involving a third population of neurons (Figure 8A).

The emergence of zero-lag synchronization through an indirect excitatory pathway has been reported in other computational work (Vicente et al., 2008); however, previous work did not address the question of how a network can transiently synchronize and desynchronize in response to stimulation. Using two different models of neuronal activity, we showed that patterns of activity in a relay network generally remain stuck in a strict limit cycle and are highly unresponsive to external stimuli. This limitation is particularly problematic given the high prevalence of relay networks in brain regions that play a central role in the integration of polysensory information, including dorsolateral prefrontal cortex, posterior cingulate cortex, and insula (Sporns et al., 2007). These regions, by their anatomical location and functional role, are expected to be highly responsive to input activation. Our simulation results provide a potential mechanism whereby a fixed anatomical substrate based on a relay network can, through selective gain inhibition, modulate its firing rate in response to an incoming stimulus. This mechanism is similar in essence to a recent gating network (Vogels and Abbott, 2009) where responses can be gated “on” by a command signal that disrupts the precise balance of excitation and inhibition. In our case, increased gain inhibition provides a way of breaking the fixed limit cycle attractor of a populations of neurons. In living systems, synapse-specific gain inhibition could be achieved by homeostatic mechanisms that dampen network reverberation, as evidenced in CA3 pyramidal cells (Kim and Tsien, 2008). It could also be achieved via cholinergic modulation, which performs cell-specific targeting and exhibits rapid response times (Ford et al., 2012; Taylor and Smith, 2012).

Zero-lag synchronization is proposed to play a number of functional roles in neuronal information processing. Synchronized activity may enhance the saliency of incoming stimuli, thus controlling the flow of information transmitted to downstream neurons. Zero-lag synchronization also provides an exquisite mechanism for precise temporal responses to rhythmic stimuli (Thivierge and Cisek, 2008, 2011), and may in itself constitute a unique channel for information transmission. Conceptually, a code based on synchronized action potentials necessitates a fewer number of presynaptic neurons to generate a postsynaptic response, and therefore allows for a greater number of input combinations than a code based on asynchronous activity (Stevens, 1994). In DS-ON ganglion cells, transient zero-lag synchronization is proposed to play a role in movement detection (Ackert et al., 2006), where a prominent synchronized/desynchronized response reinforces the presence of movement along a cell's preferred direction.

The transient synchronization of a neuronal population in response to a stimulus is supported by a range of experiments in cat cortex (Gray and Singer, 1989) as well as human electroencephalography (Rodriguez et al., 1999). A simulated network that generates synchronized oscillations only as long as a specific external signal is applied—and returns to a non-synchronized state once the signal is removed—is consistent with experiments where oscillations are observed only during the presence of a particular stimulation (Doiron et al., 2003; Ackert et al., 2006).

## Conclusion and future work

Taken together, our simulation results show that a variety of factors—including patterns of synaptic connectivity, delays in synaptic transmission, synaptic efficacies, selective gain inhibition, and surrounding network activity—contribute to both spontaneous and evoked activity in local neuronal networks. These factors provide a panoply of constraints and degrees of freedom that shape the landscape of behaviors that emerge from the interaction of neurons in synaptic circuits of the brain. Future work could extend our results by investigating how connectivity schemes (e.g., allowing both excitatory and inhibitory connections) delimit the patterns of activity produced in local populations of neurons.

### Conflict of interest statement

The authors declare that the research was conducted in the absence of any commercial or financial relationships that could be construed as a potential conflict of interest.
